# Supplementary material for: TRPM8′s Role in the Shift Between Opioid and Cannabinoid Pathways in Electroacupuncture for Inflammatory Pain in Mice
Source: Int J Mol Sci. 2024 Dec 3;25(23):13000. doi: 10.3390/ijms252313000 (PMC11641359; doi:10.3390/ijms252313000)
Supplement: Supplementary file 1 [file ijms-25-13000-s001.zip › ijms-3286388-supplementary.pdf]

Supplemental Table 1: Summary of Von Frey Test Values and Statistical Analyses

Von Frey test value, g (mean ± SD)	Baseline	CFA	Day 1	Day 2
**Figure 1C**				
Naïve	6.5 ± 0.35	6.58 ± 0.33	6.37 ± 0.16	6.38 ± 0.34
CFA	6.47 ± 0,37	1.28 ± 0.23	1.47 ± 0.22	1.53 ± 0.21
CFA+EA	6.15 ± 0.19	1.35 ± 0.21	3.12 ± 0.33	3.13 ± 0.16
CFA+sham-EA	6.55 ± 0.24	1.27 ± 0.1	1.27 ± 0.09	1.27 ± 0.09
CFA+Lido+EA	6.42 ± 0.34	1 ± 0.22	1.65 ± 0.24	1.63 ± 0.29
**Figure 2B**				
CFA+Vehicle+EA	6.5 ± 0.43	1.23 ± 0.31	2.96 ± 0.22	3.07 ± 0.24
CFA+Naloxone+EA	6.17 ± 0.39	1.34 ± 0.25	1.81 ± 0.33	1.81 ± 0.43
CFA+Naloxone+EA+AM251	6.4 ± 0.1	1.13 ± 0.13	1.37 ± 0.33	1.39 ± 0.3
CFA+AM251+EA	6.54 ± 0.27	1.14 ± 0.19	2.39 ± 0.21	2.39 ± 0.29
**Figure 3B**				
Vehicle+EA	6.08 ± 0.26	1.18 ± 0.25	3.08 ± 0.26	3.07 ± 0.27
AMTB+EA	6.33 ± 0.22	1.6 ± 0.44	2.93 ± 0.12	2.97 ± 0.31
**Figure 3C**				
AMTB+Naloxone+EA	6.37 ± 0.22	1.55 ± 0.33	3.3 ± 0.24	3.17 ± 0.32
AMTB+AM251+EA	6.46 ± 0.25	1.5 ± 0.39	1.54 ± 0.1	1.42 ± 0.23
AMTB+EA	6.33 ± 0.22	1.6 ± 0.43	2.93 ± 0.12	2.97 ± 0.31
**Figure 4A**				
CFA	6.45 ± 0.25	1.5 ± 0.2	1.57 ± 0.29	1.78 ± 0.15
CFA+EA	6.5 ± 0.14	1.37 ± 0.3	3.63 ± 0.22	3.77 ± 0.23
**Figure 4B**				
Vehicle+EA	6.37 ± 0.21	1.23 ± 0.16	3.62 ± 0.25	3.73 ± 0.10
Naloxone+EA	6.35 ± 0.3	1.33 ± 0.22	3.56 ± 0.23	3.77 ± 0.17
**Figure 4C**				
Vehicle+EA	6.4 ± 0.20	1.33 ± 0.25	3.73 ± 0.23	3.68 ± 0.13
AM251+EA	6.55 ± 0.18	1.27 ± 0.18	1.58 ± 0.26	1.63 ± 0.25
**Figure 5B**				
Menthol+EA	6.25 ± 0.43	1.37 ± 0.24	3.98 ± 0.55	3.97 ± 0.5
Vehicle+EA	6.32 ± 0.13	1.4 ± 0.15	2.83 ± 0.24	3.05 ± 0.36
**Figure 5C**				
Vehicle+EA	6.42 ± 0.22	1.32 ± 0.35	3.68 ± 0.46	3.66 ± 0.4
Menthol+EA	6.48 ± 0.22	1.30 ± 0.25	3.75 ± 0.23	3.86 ± 0.54
**Figure 6B**				
Vehicle+WIN	6.37 ± 0.18	1.15 ± 0.21	3.13 ± 0.12	3.28 ± 0.17
AM251+WIN	6.37 ± 0.47	1.27 ± 0.25	1.58 ± 0.15	1.52 ± 0.2
AMTB+WIN	6.48 ± 0.18	1.23 ± 0.14	4.03 ± 0.3	4.12 ± 0.15
**Figure 6C**				
$TRPM8^{-/-}$ Vehicle+Win	6. 27 ± 0.38	1.28 ± 0.15	4..6 ± 0.32	4.87 ± 0.35
$TRPM8^{-/-}$ AM251+Win	6.42 ± 0.18	1.27 ± 0.27	1.66 ± 0.33	2.05 ± 0.24
WT Vehicle+Win	6.42 ± 0.29	1.18 ± 0.07	3.4 ± 0.18	3.68 ± 0.42
